# Clinically accessible neuroimaging predictors of post-stroke neurocognitive disorder: a prospective observational study

**DOI:** 10.1186/s12883-021-02117-8

**Published:** 2021-02-25

**Authors:** Till Schellhorn, Eva Birgitte Aamodt, Stian Lydersen, Stina Aam, Torgeir Bruun Wyller, Ingvild Saltvedt, Mona Kristiansen Beyer

**Affiliations:** 1 Division of Radiology and Nuclear Medicine, Oslo University Hospital, Oslo, Norway; 2 Institute of Clinical Medicine, University of Oslo, Oslo, Norway; 3 Regional Centre for Child and Youth Mental Health and Child Welfare, Department of Mental Health, Faculty of Medicine and Health Sciences, Norwegian University of Science and Technology (NTNU), Trondheim, Norway; 4 Department of Neuromedicine and Movement Science, Faculty of Medicine and Health Science, Norwegian University of Science and Technology (NTNU), Trondheim, Norway; 5 Department of Geriatric Medicine, Clinic of Medicine St. Olavs Hospital, Trondheim University Hospital, Trondheim, Norway; 6 Department of Geriatric Medicine, Oslo University Hospital, Oslo, Norway

**Keywords:** Stroke imaging, Cognitive impairment, Post stroke dementia, Stroke volume, White matter lesions

## Abstract

**Background:**

Neurocognitive disorder (NCD) is common in stroke survivors. We aimed to identify clinically accessible imaging markers of stroke and chronic pathology that are associated with early post-stroke NCD.

**Methods:**

We included 231 stroke survivors from the “Norwegian Cognitive Impairment after Stroke (Nor-COAST)” study who underwent a standardized cognitive assessment 3 months after the stroke. Any NCD (mild cognitive impairment and dementia) and major NCD (dementia) were diagnosed according to “Diagnostic and Statistical Manual of Mental Disorders (DSM-5)” criteria. Clinically accessible imaging findings were analyzed on study-specific brain MRIs in the early phase after stroke. Stroke lesion volumes were semi automatically quantified and strategic stroke locations were determined by an atlas based coregistration. White matter hyperintensities (WMH) and medial temporal lobe atrophy (MTA) were visually scored. Logistic regression was used to identify neuroimaging findings associated with major NCD and any NCD.

**Results:**

Mean age was 71.8 years (SD 11.1), 101 (43.7%) were females, mean time from stroke to imaging was 8 (SD 16) days. At 3 months 63 (27.3%) had mild NCD and 65 (28.1%) had major NCD. Any NCD was significantly associated with WMH pathology (odds ratio (OR) = 2.73 [1.56 to 4.77], *p* = 0.001), MTA pathology (OR = 1.95 [1.12 to 3.41], *p* = 0.019), and left hemispheric stroke (OR = 1.8 [1.05 to 3.09], *p* = 0.032). Major NCD was significantly associated with WMH pathology (OR = 2.54 [1.33 to 4.84], *p* = 0.005) and stroke lesion volume (OR (per ml) =1.04 [1.01 to 1.06], *p* = 0.001).

**Conclusion:**

WMH pathology, MTA pathology and left hemispheric stroke were associated with the development of any NCD. Stroke lesion volume and WMH pathology were associated with the development of major NCD 3 months after stroke. These imaging findings may be used in the routine clinical setting to identify patients at risk for early post-stroke NCD.

**Trial registration:**

ClinicalTrials.gov, NCT02650531, Registered 8 January 2016 – Retrospectively registered.

**Supplementary Information:**

The online version contains supplementary material available at 10.1186/s12883-021-02117-8.

## Background

Neurocognitive disorder (NCD), which includes mild and major NCD, is common after stroke [1, 2] and a major cause of disability for stroke survivors [3, 4]. Mild and major NCD are the latest nomenclature for the previously used diagnoses “mild cognitive impairment” and “dementia”, defined in the most recent “Diagnostic and Statistical Manual of Mental Disorders (DSM-5)” [5, 6]. Reported prevalence of major NCD after a first-ever stroke ranges from 10 to 40% [2], and is stated to be around 30% for recurrent stroke [7]. Up to 71% of stroke patients are reported to have mild NCD 3 months post stroke, depending on assessment methods and diagnostic criteria used [1]. Three months after stroke, the prevalence in the “Norwegian Cognitive Impairment after Stroke (Nor-COAST)” study were found to be 29 and 26% for mild and major NCD, respectively [8]. Age, education, stroke severity, and previous stroke seem to be clinically important factors for the development of NCD, while white matter hyperintensities (WMH), medial temporal lobe atrophy (MTA) [9, 10], strategic stroke location, and total brain volume have been suggested as important imaging findings associated with the development of cognitive impairment [11, 12]. Seminal neuropathological observations by Tomlinson et al., published in 1970 [13] and backed up by a recent study [14], suggested a relationship between stroke volume, stroke location, and cognitive impairment, while another recent study found WMH-volume and not the stroke volume to be a significant predictor of cognitive impairment after a mild stroke [15]. While WMHs are consistently stated as strong predictors of post-stroke cognitive impairment, the importance of the stroke lesion volume is still disputable [16], especially for small stroke volumes (~ 5 ml) that are commonly observed in the clinical routine [17].

In order to be clinically feasible, neuroimaging markers should be accessible on routine acute stroke imaging, not only on advanced research sequences. Fast routine stroke imaging combined with visual rating scales would allow for early clinical predictions of post-stroke NCD without the need for complicated MRI post-processing software and advanced sequences. The clinical translation of this study is the identification of robust robust neuroimaging markers of post-stroke NCD, which will help to estimate the risk of post-stroke cognitive impairment for the individual patients and allow the identification of patients susceptible to preventive interventions.

The aim of this study was to identify clinically accessible neuroimaging markers that are associated with early post-stroke NCD.

Our hypothesis was that patients with larger stroke volume, left-sided stroke, strategic stroke locations, higher WMH burden and higher MTA-score more frequently develop post-stroke NCD.

## Methods

### Nor-COAST

The current study uses data from the Norwegian Cognitive Impairment After Stroke (Nor-COAST) study; a prospective longitudinal multicenter cohort study recruiting patients hospitalized with acute stroke in five Norwegian stroke units from May 2015 to March 2017. Details of the Nor-COAST study are described by Thingstad et al. [18].

### Population

All patients admitted to the five stroke units participating in Nor-COAST with suspected stroke were screened for inclusion. A stroke was diagnosed according to the World Health Organisation (WHO) criteria [19] as a focal (or at times global) neurological impairment of sudden onset, and lasting more than 24 h (or leading to death) and of presumed vascular origin or an findings of acute infarction or intra-cerebral hemorrhage on MRI.

Eligible patients for the inclusion in Nor-COAST were according to the inclusion criteria (a) admitted with acute ischemic or hemorrhagic stroke hospitalized within 1 week after onset of symptoms; (b) age over 18 years; and (c) fluent in a Scandinavian language. Exclusion criteria were (a) symptoms explained by other disorders than ischemic brain infarct or intracerebral hemorrhages; (b) expected survival less than 3 months after stroke based on a clinical assessment by experienced stroke physicians. Patients with pre-existing cognitive impairment or dementia were not excluded. Inclusion criteria for the MRI substudy were (a) patient included in Nor-COAST; (b) modified Rankin scale < 5 before the stroke; (c) able to cooperate during MRI. Exclusion criteria for MRI were (a) severe functional impairment making MRI impossible to perform; (b) medical contraindications for MRI like claustrophobia or pacemaker; (c) patient declined participation in MRI substudy. Participation in the MRI substudy was optional.

### MRI acquisition

A study-specific brain MRI was performed as early as possible during the acute/subacute phase of the stroke. Brain scans were acquired at all five participating hospitals, using one specific MRI-scanner on each site (GE Discovery MR750, 3 T; Siemens Biograph_mMR, 3 T; Philips Achieva dStream, 1.5 T; Philips Achieva, 1.5 T; Siemens Prisma, 3 T). The study protocol consisted of 3D-T1 weighted, axial T2, 3D-Fluid attenuated inversion recovery (FLAIR), diffusion-weighted imaging (DWI), and susceptibility-weighted imaging (SWI). Details about the MRI protocol have previously been described [20].

### Image analysis

#### Coregistration and stroke segmentation

The 3D-T1 sequence was linearly registered to the 1 mm “Montreal Neurological Institue-152” (MNI-152) T1 template with the help of “Oxford Centre for Functional Magnetic Resonance of the Brain (FMRIB)” version 6.0 [21] Linear Image Registration Tool (FLIRT) [21, 22]. DWI trace images were coregistered and resampled to the already co-registered 3D-T1 using the same software (FLIRT).

The stroke lesion volume was defined as equivalent to the ischemic core that represents the amount of irreversibly destroyed brain parenchyma, identified as diffusion restriction on the DWI sequence. Acute infarcts were semi-automatically labeled with the help of the “Insight Segmentation and Registration Toolkit-Snap (ITK-Snap)” snake tool (v. 3.8.0) [23] in order to create lesion masks. Hemorrhagic stroke lesions were included in this analysis. We did not split the analysis of stroke lesion volume in ischemic and hemorrhagic stroke because of the small number and comparable volume of hemorrhagic strokes. Lesion masks were created for all patients who had visible diffusion restriction on DWI. The masked stroke volume in mm^3^ was automatically measured by ITK-snap (v. 3.8.0), exported to a comma separated values (CSV) file, and converted to milliliters (ml).

#### Localization of acute stroke

Infarcts in the thalamus, angular gyrus, hippocampus, parahippocampal gyrus, caudate, substantia nigra, red nucleus, lateral and medial globus pallidus were considered strategic infarcts in accordance with previous findings [24, 25]. Coordinates of masked stroke lesions were used to identify the hemisphere and corresponding anatomical structure in Talairach brain atlases [26]. The anatomical atlas labels were retrieved with the help of the “AtlasReader” python package [27].

#### Visually rated variables

WMH of presumed vascular origin were classified according to the widely used Fazekas scale [28]. The acute stroke lesion was not included in the Fazekas score. Fazekas grade of 1 was considered normal for all ages. The following Fazekas grades were used to identify WMH pathology. Fazekas grade 2 was considered normal in patients 71 years or older, whereas Fazekas grade 3 was always regarded as pathological [29]. MTA was assessed according to the established MTA scale [30]. Findings were considered pathological where the mean MTA of both sides was ≥1.5 under the age of 75, a value ≥2 below the age of 85, and a value of ≥2.5 below 95 years, as recommended by Ferreira et al. [31]. For a thorough description of the methods of visual assessment please see Schellhorn et al. [20]. Please see Supplement A4 for a description of the Fazekas scale, and MTA scale.

#### Clinical characteristics

Demographic and clinical variables were recorded at the time of the index stroke by study nurses and stroke physicians. The following data were included: Age, years of education, gender, stroke severity measured with the National Institute of Health Stroke Scale (NIHSS), hypertension, hypercholesterolemia, smoking, diabetes mellitus, clinical history of previous stroke and clinical stroke subtype according to the “Trial of ORG 10172 in Acute Stroke Treatment (TOAST)” classification [32].

#### Cognitive assessment

Pre-stroke cognitive impairment was assessed by interviews of relatives or caregivers, using the Global Deterioration Scale (GDS). The GDS ranges from 1 to 7. A GDS score of 3 or above is categorized as pathological [33], with 3 representing mild NCD and values from 4 to 7 are representing major NCD.

The diagnosis of post-stroke NCD was based on the “Diagnostic Statistical Manual of Mental Disorders (DSM5)” implementing neuropsychological test scores. Five of six cognitive domains cited in the DSM-5 were assessed with a domain specific test: perceptual-motor function, language, executive function, learning and memory, and complex attention. Social cognition was not measured. The perceptual-motor function was tested with the visuospatial/executive domain from MoCA. Language was evaluated with the verbal fluency test - category (animals). Executive function was measured with the trail making test B and verbal fluency test – letter (FAS). Learning and memory was evaluated with the word list recall test. Complex attention was measured with the trail making test -A [8].

Patients scoring < − 1.5 SD in at least one cognitive domain were defined as having poststroke NCD. According to these criteria the cognitive status after 3 months was classified into normal cognition, mild NCD or major NCD. All patients with either mild or major NCD were classified as any NCD.

Major NCD was defined as post stroke NCD and dependency in I-ADL; whereas mild NCD was defined as post stroke NCD with independence in I-ADL, as previously published [8]. Moreover, cognitive status during hospital stay and after 3 months was additionally assessed with the “Montreal Cognitive Assessment Test (MoCA) [34], which is a comprehensive screening test used to detect NCD.

### Statistical analysis

In order to perform logistic regression analyses, the three cognitive outcome categories were dichotomized in two different ways: (a) normal cognition versus any NCD; and (b) major NCD versus normal cognition or mild NCD. This allowed us to separately analyze predictors of any NCD and major NCD. We attempted ordinal logistic regression with three possible cognitive outcomes (normal, mild NCD and major NCD) as the dependent variable. However, the ordinal logistic model turned out to be unsuited for our study as the odds ratios differed considerably between the thresholds.

The predictor variables stroke volume, strategic infarct, affected hemisphere, MTA pathology, and WMH pathology were analyzed one at a time for any NCD and major NCD using univariate logistic regression and all together in one multiple logistic regression model. We analyzed further if the pre-stroke cognitive status influenced the effect of the investigated predictor variables. For this purpose, the logistic regression analyses were repeated with pre-stroke GDS included in the logistic regression model.

All results were adjusted for the known important confounders age, sex, and education if not explicitly marked as unadjusted. Missing values were handled using available case analysis, that is, each analysis includes all the cases with available data on the variables in that analysis. Two-sided *p*-values < 0.05 were considered as indicators of statistical significance, and we reported 95% confidence intervals (CI), where relevant. Statistical analyses were carried out using the software Stata 16 [35].

## Results

### Study population

**Fig. 1:**
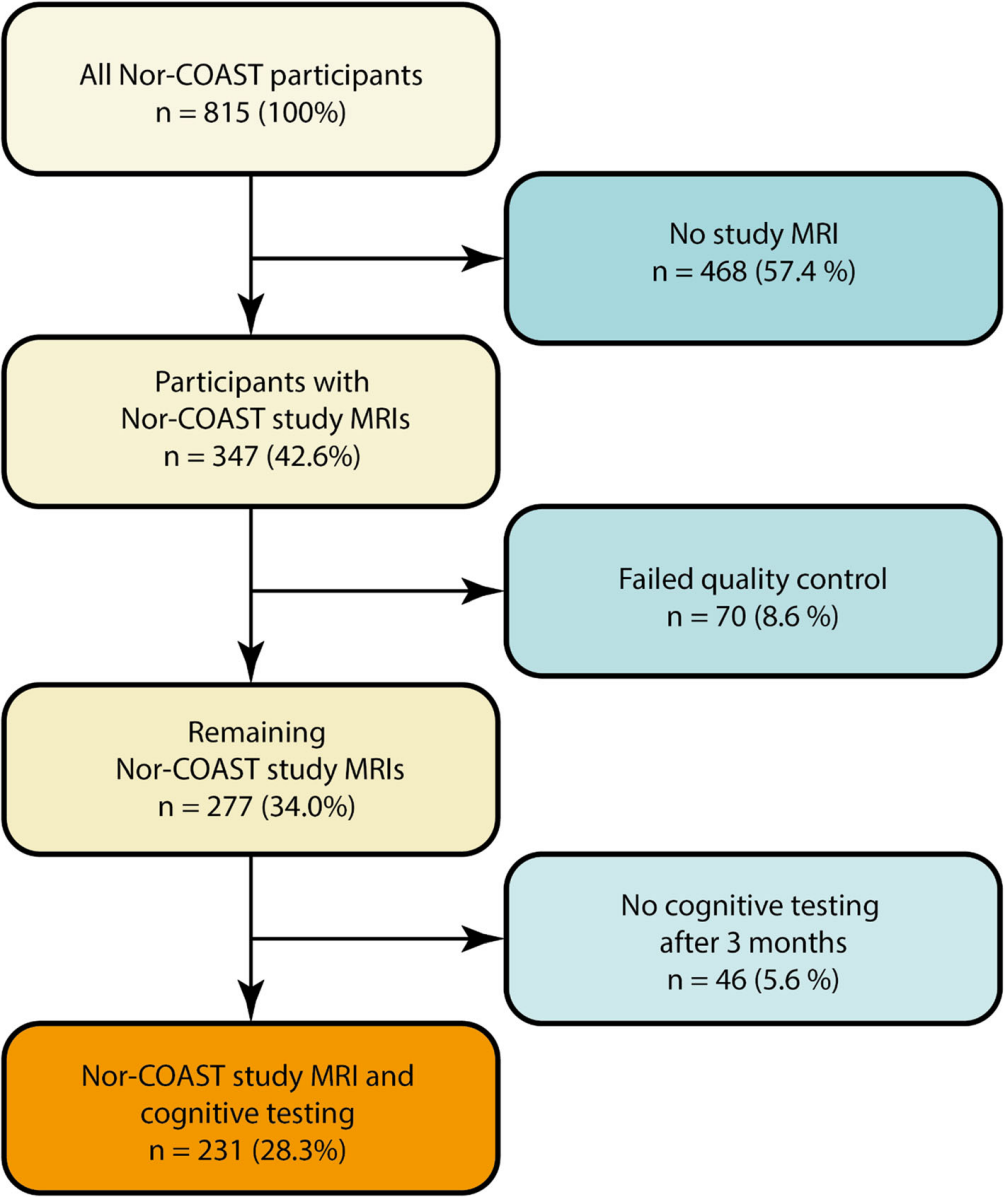
Study population overview

The total Nor-COAST study population consisted of 815 participants. Study specific MRI scans that fulfilled all quality requirements were performed in 347 (42.6%) of all Nor-COAST participants. The remaining 468 (57.4%) were not eligible for this MRI study due to severe functional impairment, not able to cooperate, medical contraindication, incomplete or inadequate MRI-protocol, or because the patient declined participation in the MRI sub study. Of the 347 patients with study specific MRI scans 70 failed quality control due to technical reasons, movement or missing sequences. Cognitive status according to DSM-5 criteria was performed in 231 (66.6%) patients 3 months after the stroke, resulting in a study sample of 231. Participation in this study is shown in Fig. 1 below. The mean (SD) time to hospital admission was 13 [27] hours. The mean (SD) time to MRI was 8 [16] days.

For the whole study sample at baseline the mean age (SD) of patients was 71.8 (11.1), 101 (43.7%) were female, mean length of education was 12.3 (3.7) years, 32 (13.6%) had a history of previous clinical stroke. The mean (SD) MoCA score was 23.1 (3.1) 3 month after stroke. Stroke subtype according to TOAST classification (*n* = 208): Large-vessel disease 18 (8.7%), cardioembolic disease 44 (21.2%), small-vessel disease 59 (28.4%), other etiology 4 (1.9%), undetermined etiology 83 (39.9%). A hemorrhagic stroke was identified in 17 (7.4%) patients. Mild pre-stroke NCD was evident in 13 (5.6%) and major pre-stroke NCD in 10 (4.4%) of the patients. A complete frequency table of GDS grades versus post-stroke cognitive outcome can be found in the Supplemental Table A1. Clinical characteristics grouped by cognitive outcome are summarized in Table 1.

**Table 1:** Baseline characteristics in the cognitive outcome groups 3 months after index stroke

	Normal cognition	Mild NCD^c^	Major NCD	Any NCD	Total
	***n*** = 103	***n*** = 63	***n*** = 65	***n*** = 128	***n*** = 231
**Female - n (%)**	46 (44.7)	23 (36.5)	32 (49.2)	55 (43.0)	101 (43.7)
**Age (years) - mean (SD)**	69.4 (11.2)	69.7 (6.7)	77.5 (10.3)	73.6 (10.7)	71.8 (11.1)
**Education (years) - mean (SD)**	13.2 (3.8)	12.7 (3.5)	10.5 (3.2)	11.6 (3.5)	12.3 (3.7)
**Time (hrs) to admission – mean (SD)**	11 (27)	10 (16)	19 (34)	15 (27)	13 (27)
**Hypertension - n (%)**	43 (41.8)	31 (49.2)	35 (53.9)	66 (51.6)	109 (47.2)
**Diabetes mellitus - n (%)**	13 (12.6)	10 (15.9)	16 (24.6)	26 (20.3)	39 (16.9)
**Hypercholesterolemia - n (%)**	33 (32.0)	28 (44.4)	29 (44.6)	57 (44.5)	90 (39.0)
**Smoker - n (%)**					
**previous**	36 (35.0)	24 (38.1)	30 (46.2)	54 (42.2)	90 (39.0)
**current**	25 (24.3)	13 (20.3)	11 (16.9)	24 (18.8)	49 (21.2)
**NIHSS**^a^ **score - mean (SD)**	3.1 (3.8)	3.1 (2.4)	5.2 (6.7)	4.2 (5.2)	3.7 (4.6)
**Previous clinical stroke - n (%)**	11 (10.7)	7 (11.1)	14 (21.5)	21 (16.4)	32 (13.6)
**MoCA**^**b**^ **- mean (SD)**	26.3 (2.7)	24.5 (3.1)	19.3 (5.4) ^(*n* = 64)^	22.2 (5.0) ^(*n* = 127)^	23.1 (5.1) ^(*n* = 230)^

^a^*NIHSS* National Institute of Health Stroke Scale. ^**b**^*MoCA* “Montreal Cognitive Assessment Test”, scored at 3 months. ^c^*NCD* Neurocognitive disorder

### MRI

#### Imaging markers

**Fig. 2:**
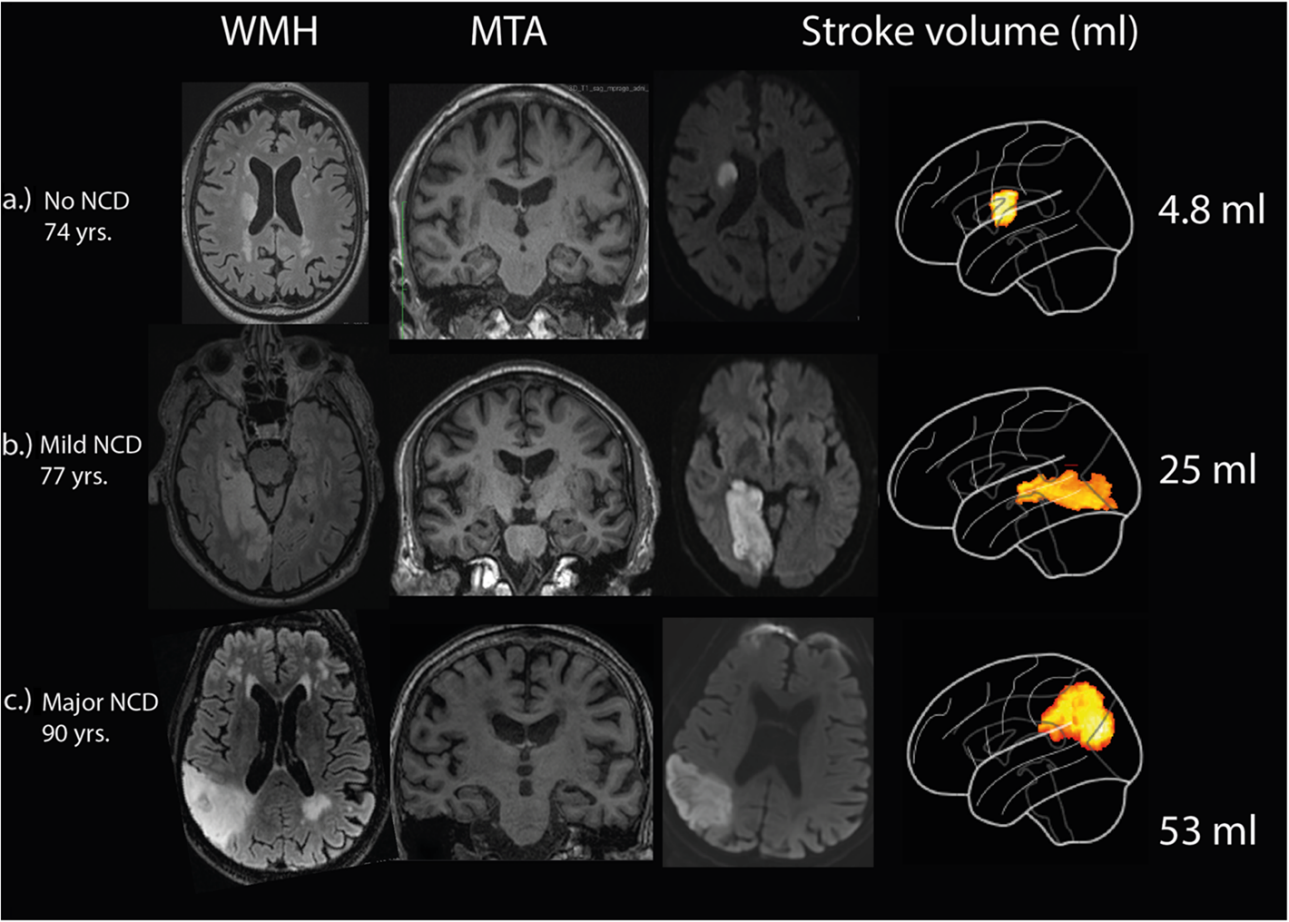
Examples of WMH, MTA and stroke lesion volume for normal cognition, mild and major NCD. **a**.) Normal cognitive status after 3 months with normal MTA (medial temporal lobe atrophy) and WMH (white matter hyperintensity) score and an average stroke volume for this group; **b**.) mild NCD (neurocognitive disorder) with a relatively small stroke volume, pathologic MTA and pathologic WMH score; **c**.) Major NCD with a larger stroke volume, normal MTA and pathologic WMH score

The mean (SD) stroke volume was 8.7 (17.4) ml, the left hemisphere was affected in 102 (45.1%) of the patients, both hemispheres in 6 (2.7%), 23 (9.9%) had suffered a strategic stroke. An overview of all affected locations is given in Supplemental Table A2. Pathologic WMH score was found in 90 (39%) and 82 (35.5%) had pathologic MTA score. Imaging findings grouped by cognitive outcome are summarized in Table 2. Imaging examples of WMH, MTA and stroke lesion volume for the three cognitive outcomes are given in Fig. 2.

**Table 2:** Baseline imaging findings according to cognitive outcome groups 3 months after acute stroke

	Normal cognition ***n*** = 103	Mild NCD^d^ ***n*** = 63	Major NCD ***n*** = 65	Any NCD ***n*** = 128	Total ***n*** = 231
**Stroke volume (ml) - mean (SD)**	7 (10.8)	4.1 (7)	16.1 (27.7)	10.2 (21.2)	8.7 (17.4)
**Stroke volume (ml) – median (IQR)**^**a**^	1.7 (0.5–9.1)	1.7 (0.5–3.7)	2.9 (0.7–21-1)	1.9 (0.6–7.5)	1.8 (0.2–5.3)
**Hemorrhagic stroke - n (%)**	5 (4.6)	7 (11.1)	5 (7.7)	12 (9.4)	17 (7.4)
**Left hemisphere – n (%)**	38 (37.6)	33 (55)	31 (47.7)	64 (51.2)	102 (45.1)
**Both hemispheres – n (%)**	2 (2)	3 (5)	1 (1.5)	4 (3.2)	6 (2.7)
**Strategic infarct - n (%)**	10 (9.7)	6 (9.5)	7 (10.8)	13 (10.2)	23 (9.9)
**Pathologic WMH**^b^ **score - n (%)**	27 (26.2)	25 (39.7)	38 (58.5)	63 (49.2)	90 (39)
**Pathologic MTA**^**c**^ **score - n (%)**	28 (27.2)	27 (42.9)	27 (41.5)	54 (42.2)	82 (35.5)

^a^*IQR* Inter quartile range, ^b^*WMH* White matter hyperintensities. ^c^*MTA* Medial temporal lobe atrophy. ^d^*NCD* Neurocognitive disorder

#### Cognitive outcome after 3 months

Three months after the stroke 128 (55.4%) had any NCD, mild NCD was found in 63 (27.3%) and major NCD in 65 (28.1%).

#### Any NCD

Any NCD after 3 months was significantly associated with WMH pathology (odds ratio (OR) = 2.73 [1.56 to 4.77]), MTA pathology (OR = 1.95 [1.12 to 3.41]) and left hemispheric stroke (OR = 1.8 [1.05 to 3.09]). The resulting ORs remained approximately constant after adjusting for age, sex, education (see Table 3). Further inclusion of pre-stroke cognitive status and subsequently all predictor variables (stroke volume, WMH pathology, MTA pathology, left hemispheric stroke and strategic infarct) in the multivariate logistic regression resulted in persistent robust ORs (see Supplemental Table A3).

**Table 3:** Results from logistic regression analyses with any or major neurocognitive disorder as outcome variable

Imaging marker		n	Any NCD OR [95%CI]	***p***-value	Major NCD OR [95%CI]	***p***-value
Stroke lesion volume (ml)	Unadjusted	231	1.01 [0.99 to 1.03]	0.176	1.04 [1.02 to 1.06]	0.001
	Adjusted^a^	231	1.01 [0.99 to 1.03]	0.275	1.04 [1.01 to 1.06]	0.001
WMH pathology	Unadjusted	231	2.73 [1.56 to 4.77]	0.001	3.09 [1.71 to 5.58]	0.001
	Adjusted^a^	231	2.37 [1.33 to 4.23]	0.003	2.54 [1.33 to 4.84]	0.005
MTA pathology	Unadjusted	231	1.95 [1.12 to 3.41]	0.019	1.43 [0.79 to 2.59]	0.231
	Adjusted^a^	231	2.13 [1.18 to 3.87]	0.013	1.87 [0.95 to 3.68]	0.069
Left hemispheric stroke	Unadjusted	226	1.80 [1.05 to 3.09]	0.032	1.12 [0.63 to 2.01]	0.693
	Adjusted^a^	226	1.74 [1 to 3.04]	0.051	1.01 [0.53 to 1.93]	0.966
Strategic infarct	Unadjusted	231	1.05 [0.44 to 2.51]	0.91	1.13 [0.44 to 2.89]	0.796
	Adjusted^a^	231	1 [0.4 to 2.48]	1	1.17 [0.41 to 3.3]	0.770

The Odds Ratio (OR) of any NCD or major NCD in the presence of a defined imaging marker is shown in the table above. ORs are reported unadjusted and ^a^adjusted for age, sex, and education

#### Major NCD

Major NCD after 3 months was significantly associated with stroke lesion volume (OR = 1.04 [1.02 to 1.06]) and WMH pathology (OR = 3.09 [1.71 to 5.58]). The resulting ORs remained approximately constant after adjusting for age, sex, education (see Table 3). Further inclusion of pre-stroke cognitive status and subsequently all predictor variables (stroke volume, WMH pathology, MTA pathology, left hemispheric stroke and strategic infarct) in the multivariate logistic regression resulted in persistent robust ORs (see Supplemental Table A3).

A comparison of our findings compared to those of other studies investigating the relationship between neuroimaging biomarkers and cognitive impairment is summarized in Table 4.

**Table 4:** Comparison of our results with the literature

Study	Study population n	Follow-up Time months	Results	Conclusion
**Stroke volume**				
Puy [14]	365	6	Mean (SD) stroke volume **7.6 (23.121)**	Increasing stroke volume is associated with lower Global Cognitive Score
Munsch [24]	428	3	Median [IQR] stroke volume (ml) for good outcome **6 [0–196]** Median [IQR] stroke volume (ml) for poor outcome **17 [0–211]**	Higher stroke volume is associated with lower MoCA score
Jokinen [9]	560	36	Mean (SD) stroke volume (ml) **25.7 (39.8)** Stroke volume affected Trail Making, Stroop dots and verbal fluency among others	Estimated total infarct volume is associated with specific cognitive deficits
Nor-COAST	231	3	Mean (SD) stroke volume (ml) for mild NCD **4.1 (7)** Mean (SD) stroke volume (ml) for major NCD **16.1 (27.7)**	Increasing stroke volume is associated with major NCD
**White matter hyperintensities (WMH)**				
Puy [14]	365	6	Median [IQR] WMH score **1 [1–2]**	WMH were not associated with Global Cognitive Score
Jokinen [9]	560	36	WMH significantly predicted Trail Making time, verbal fluency and visual reproduction among others.	WMH are associated with specific cognitive deficits post-stroke
Molad [16]	397	24	WMH affected Global Cognitive Score, memory score and executive function score among others	WMH burden was associated with poor post-stroke cognitive performance
Nor-COAST	231	3	Any NCD group: 49.2% pathological WMH Major NCD group: 58.5% pathological WMH	WMH pathology is significantly associated with any and major post-stroke NCD
**Medial temporal lobe atrophy (MTA)**				
Puy [14]	365	6	Median [IQR] MTA 2 [0–3]	MTA a week determinant of Global Cognitive Score
Jokinen [9]	560	36	MTA is associated with processing speed, executive function, and memory	MTA is a strong predictor of cognitive performance
Firbank [36]	79	3	Mean (SD) MTA score for no dementia: 2.6 (1.8) Mean (SD) MTA score for dementia: 3.1 (1.9)	MTA is the strongest predictor of memory function post-stroke
Nor-COAST	231	3	Normal cognition group: 27.2% pathological MTA Any NCD group: 42.2% pathological MTA	MTA is associated with any NCD
**Left hemispheric stroke**				
Puy [14]	365	6	Percantage of patients with left hemispheric stroke: 47%	Left hemispheric stroke was moderately associated with Global Cognitive Score
Dienanta [37]	32	0.5	Normal MMSE Score left hemispheric stroke: 53% Abnormal MMSE Score lef hemispheric stroke: 47%	Left hemispheric stroke was not associated with post-stroke cognitive outcome
Sagnier [38]	265	3	Language, abstraction, and delayed recall performances were associated with left sided stroke	Left sided stroke was associated with cognitive impairment
Nor-COAST	231	3	Percentage of left hemispheric stroke in normal post-stroke cognition group: 37.6% Percantage of left hemispheric stroke in any NCD group: 51.2%	Left hemispheric stroke was not associated with mild or major NCD
**Stratgic stroke**				
Puy [14]	365	6	Percantage of patients with strategic strokes: 25%	Strategic strokes were strongly associated with Global Cognitive Score
Munsch [24]	428	3	Median (range) number of eloquent voxels in good outcome group: 25 (0–821) Median (range) number of eloquent voxels in poor outcome group: 138 (0–13,359)	Strategic strokes were significantly associated with poor cognitive outcome (MoCA)
Zhao [25]	410	3 to 6	Infarcts in left basal ganglia, left and right frontal, left parietal and left occipital influenced the MoCA score most	Strategic infarcts were associated with MoCA score
Nor-COAST	231	3	Percantage of strategic infarcts in normal cognition group: 9.7% Percantage of strategic infarcts in mil NCD: 9.5% Percantage of strategic infarcts in any NCD: 10.2%	Strategic infarcts were not associated with mild or major NCD

## Discussion

In this prospective observational cohort study of stroke survivors, we analyzed the associations between neuroimaging findings in the acute phase and NCD 3 months after the index stroke. Any NCD was significantly associated with WMH pathology, MTA pathology and left hemispheric stroke. Major NCD was significantly associated with stroke lesion volume and WMH pathology.

The fraction of patients in our study with major NCD was 28.1%, which is in line with published prevalences that range from 7.4% in population-based studies to 41.3% in hospital-based studies. The wide range of prevalence is explained by varying diagnostic criteria [39].

Our results indicate that the stroke lesion volume is a significant predictor of major NCD, even though the mean stroke lesion volume is small. For every ml the stroke volume increases, the OR of major NCD increases by 1.04. This is consistent with another study with mainly small stroke lesion volumes (mean 7.6 ml) [14], where the authors reported a significant, but small proportion of the cognitive performance to be explained by the stroke size. Three studies with mean initial stroke volume above 50 ml showed that when the initial stroke lesion volume is larger, the stroke lesion volume becomes a very strong predictor of clinical outcome measured by the modified Rankin score [40–42]. These reports are consistent with our findings. Since the odds of major NCD increases with every ml lesion volume, a small stroke lesion contributes to favorable clinical outcome after endovascular stroke therapy [43], especially when the WMH burden is low [44]. Thus, reducing the stroke lesion volume is important for reducing the odds of major NCD.

WMH pathology was in our study associated with any NCD and major NCD 3 months after stroke. This is in accordance with other studies which reported an association between WMH burden and cognitive symptoms [45–48]. The presence of severe WMH at baseline doubled the future risk of stroke and increased the risk of dementia four-fold in the “Framingham Offspring Study” [49]. Other studies confirm that WMHs are strongly associated with cerebrovascular disease [50], vascular risk factors, and dementia [49, 51, 52]. The underlying pathology is believed to reflect demyelination and axonal loss related to chronic ischemia caused by small vessel disease (SVD) [53]. SVD, for which WMH is an imaging marker, has recently been linked to glymphatic dysfunction. This glymphatic dysfunction may lead to a progression of neurodegenerative dementias in the same patients at risk for vascular dementia [54, 55] and it might explain the overlap between these entities.

We found a statistically significant positive association between MTA pathology and any NCD. This has been shown to varying degrees in previous studies [9, 14]. While MTA pathology mostly occurs in the context of neurodegenerative disease [56], it has also been associated with pure vascular cognitive impairment [57, 58]. High grades of MTA are strongly associated with dementia, especially Alzheimer’s disease [56]. Fiford et al. on the other hand have suggested that vascular damage, like WMH, is associated with greater atrophy of the hippocampus compared to controls [59]. Consequently, MTA pathology in a stroke cohort might be related to SVD as well as neurodegeneration.

We propose that stroke lesion volume and WMH pathology are important predictors for the development of major NCD 3 months after an acute stroke. An underlying mechanism might be that an acute stroke, even with a small lesion volume, unmasks the effects of reduced brain resilience as indicated by the presence of WMHs and MTA, and triggers the development of any NCD [60]. This makes the use of imaging markers as predictors valuable and suggests that reducing stroke size may prevent the early development of post-stroke NCD. The WMH pathology and MTA pathology may be modifiable by targeting risk factors for SVD, i.e. hypertension [61, 62]. Strategic infarcts were not frequent in our study. This may explain why we did not find an association with NCD.

Although the study sample in the present study is quite representative for the general stroke population [63], a weakness of our study was that the most severely affected stroke patients were not included, which makes the results most relevant for patients with moderate to mild stroke and smaller stroke lesions.

Another weakness was the relatively low number of included brain MRIs were 231, which represented 67% of the Nor-COAST study MRIs and 28% of the total Nor-COAST cohort, either due to the lack of imaging or cognitive testing. This highlights the difficulty to recruit elderly patients to longitudinal neuroimaging and cognitive testing, even though the imaging protocol was significantly shortened compared to our pilot study. To increase the imaging rate, future studies should favor analyses based on standard clinical protocols or incorporate new methods for MRI sequence acceleration and reconstruction. This would also reduce a selection bias towards recruiting participants with lower NIHSS scores. A more comprehensive neuropsychological test-battery would have been preferable but was not feasible in this elderly stroke cohort. Finally short follow-up of only 3 months after stroke could perhaps have been even better. However, no significant difference has been shown between the results of 3 and 18 months post-stroke cognitive testing in Nor-COAST [64].

A strength of our study is the prospective inclusion of acute stroke patients, which were scanned with a time effective imaging protocol adapted to an older stroke cohort. This imaging protocol and the used neuroimaging markers in our study could be implemented in clinical practice.

The identified brain pathologies associated with post-stroke NCD are straightforward to assess on clinical brain-MRIs in the form of WMH-burden and MTA-score. WMH and MTA are visual scores that can be evaluated readily by neuroradiologists or experienced clinicians in a routine stroke setting. While multiple tools have been developed for the volumetric WMH-assessment, they are still almost exclusively available in a research setting. It is of importance to differentiate WMH related to healthy ageing from pathological WMH, as done in our study. Another strength is the use of a neuropsychological assessment that is based on DSM-5 and evaluated for the Nor-COAST stroke cohort [8]. According to the DSM-5 criteria, impairment any cognitive domain is sufficient to diagnose NCD, and memory impairment (which is more appropriate for the diagnosis of Alzheimer’s disease), is not mandatory. As such, the DSM-5 criteria help to obtain robust and reproducible cognitive assessments in stroke cohorts.

## Conclusions

Development of major and any NCD 3 months after stroke is associated with acute stroke lesion volume and WMH pathology. MTA pathology is associated with any NCD. Thus, it might be possible to assess the risk of post-stroke NCD development based on clinically accessible stroke imaging. The current acute stroke therapy aims to reduce the stroke volume, and this seems to be a good strategy, not only for reducing physical disability, but also the development of major NCD in stroke survivors.

## Supplementary Information

**Additional file 1: Supplemental Table A1.** Frequencies of pre-stroke GDS-scores in every cognitive outcome group.

**Additional file 2: Supplemental Table A2.** Counts of identified stroke locations.

**Additional file 3: Supplemental Table A3.** Results from adjusted and full logistic regression analyses with neurocognitive disorder (NCD) as dependent variable.

**Additional file 4: Supplement A4.** Definitions of clinical diagnoses, MTA, and Fazekas scale.

## Data Availability

The current dataset cannot be made publicly available due to Norwegian legal restrictions A portion of data can be made available upon request to interested, qualified researchers provided that an agreement is made up. The minimal data set will enable replication of the reported study findings. Requests to access the datasets should be directed to [Mona K Beyer, monbey@ous-hf.no]”.

## Abbreviations

NCD: Neurocognitive disorder
Nor-COAST: Norwegian Cognitive impairment After Stroke study
DSM-5: Diagnostic and Statistical Manual of Mental Disorders version 5
MRI: Magnetic resonance imaging
WMH: White matter hyperintensities
MTA: Medial temporal lobe atrophy
SD: Standard deviation
OR: Odds ratio
WHO: World Health Organization
T: Tesla
FLAIR: Fluid attenuated inversion recovery
DWI: Diffusion-weighted imaging
SWI: Susceptibility-weighted imaging
MNI: Montreal Neurological Institute
FMRIB: Oxford Centre for functional magnetic resonance imaging of the brain
FLIRT: FMRIB’s Linear image registration tool – linear and intra-model registration
ITK: Insight segmentation and registration toolkit
CSV: Comma separated values
NIHSS: National Institute of Health Stroke Scale
TOAST: Trial of ORG 10172 in acute stroke treatment
GDS: Global deterioration scale
I-ADL: Instrumental activities of daily living
MoCA: Montreal cognitive assessment test
CI: Confidence interval
IQR: Inter quartile range
SVD: Small vessel disease
