# Supplementary material for: Clinically accessible neuroimaging predictors of post-stroke neurocognitive disorder: a prospective observational study
Source: BMC Neurol. 2021 Feb 25;21:89. doi: 10.1186/s12883-021-02117-8 (PMC7905565; doi:10.1186/s12883-021-02117-8)
Supplement: Supplementary file 1 — Additional file 1: Supplemental Table A1. Frequencies of pre-stroke GDS-scores in every cognitive outcome group. [file 12883_2021_2117_MOESM1_ESM.docx]

Supplemental table A1: Frequencies of pre-stroke GDS-scores in every cognitive outcome group.

|  | Normal cognition  n = 103 | Mild NCD^2^  n = 63 | Major NCD  n = 65 | Any NCD  n = 128 | Total  n = 231 |
| --- | --- | --- | --- | --- | --- |
| Pre-stroke GDS^1^ 1 (%) | 88 (85.4) | 49 (77.8) | 28 (43.1) | 77 (60.2) | 165 (71.4) |
| Pre-stroke GDS^1^ 2 (%) | 14 (13.6) | 13 20.6) | 16 (24.6) | 29 (22.7) | 43 (18.6) |
| Pre-stroke GDS^1^ 3 (%) | 1 (1.0) | 1 (1.6) | 11 (16.9) | 12 (9.4) | 13 (5.6) |
| Pre-stroke GDS^1^ 4 (%) | 0 (0.0) | 0 (0.0) | 5 (7.7) | 5 (3.9) | 5 (2.2) |
| Pre-stroke GDS^1^ 5 (%) | 0 (0.0) | 0 (0.0) | 5 (7.7) | 5 (3.9) | 5 (2.2) |
| Pre-stroke GDS^1^ 6 (%) | 0 (0.0) | 0 (0.0) | 0 (0.0) | 0 (0.0) | 0 (0.0) |
| Pre-stroke GDS^1^ 7 (%) | 0 (0.0) | 0 (0.0) | 0 (0.0) | 0 (0.0) | 0 (0.0) |

Absolute and relative frequencies of ^1^pre-stroke GDS scored at baseline grouped by post-stroke cognitive outcome. ^2^NCD = neurocognitive disorder.
