# Supplementary material for: Clinically accessible neuroimaging predictors of post-stroke neurocognitive disorder: a prospective observational study
Source: BMC Neurol. 2021 Feb 25;21:89. doi: 10.1186/s12883-021-02117-8 (PMC7905565; doi:10.1186/s12883-021-02117-8)
Supplement: Supplementary file 2 — Additional file 2: Supplemental Table A2. Counts of identified stroke locations. [file 12883_2021_2117_MOESM2_ESM.docx]

Supplemental table A2: Counts of identified stroke locations

Sub-Gyral 189

Background 139

Extra-Nuclear 64

Precentral Gyrus 49

Postcentral Gyrus 47

Precuneus 34

Middle Frontal Gyrus 33

Culmen 31

Middle Temporal Gyrus 29

Declive 25

Inferior Parietal Lobule 24

Cingulate Gyrus 24

Middle Occipital Gyrus 23

Lingual Gyrus 21

Thalamus 20

Superior Parietal Lobule 19

Cuneus 19

Inferior Temporal Gyrus 17

Superior Temporal Gyrus 17

Insula 15

Fusiform Gyrus 14

Medial Frontal Gyrus 14

Superior Frontal Gyrus 10

Lentiform Nucleus 10

Caudate 9

Pyramis 8

Subcallosal Gyrus 6

Uvula 6

Cerebellar Tonsil 6

Lateral Ventricle 6

Paracentral Lobule 5

Supramarginal Gyrus 5

Tuber 5

Inferior Frontal Gyrus 4

Angular Gyrus 4

Parahippocampal Gyrus 4

Inferior Semilunar Lobule 3

Superior Occipital Gyrus 3

Inferior Occipital Gyrus 1

Transverse Temporal Gyrus 1

Fastigium 1

Counts of all identified stroke lesion locations according to the Talairach brain atlas
