# Supplementary material for: Clinically accessible neuroimaging predictors of post-stroke neurocognitive disorder: a prospective observational study
Source: BMC Neurol. 2021 Feb 25;21:89. doi: 10.1186/s12883-021-02117-8 (PMC7905565; doi:10.1186/s12883-021-02117-8)
Supplement: Supplementary file 3 — Additional file 3: Supplemental Table A3. Results from adjusted and full logistic regression analyses with neurocognitive disorder (NCD) as dependent variable. [file 12883_2021_2117_MOESM3_ESM.docx]

Supplemental table A3: Results from adjusted and full logistic regression analyses with neurocognitive disorder (NCD) as dependent variable.

| Imaging marker |  | N | Any NCD  OR [95%CI] | p-value | Major NCD  OR [95%CI] | p-value |
| --- | --- | --- | --- | --- | --- | --- |
| Stroke volume  (ml) | Unadjusted^1^ | 231 | 1.01 [0.99 to 1.03] | 0.176 | 1.04 [1.02 to 1.06] | 0.001 |
|  | Adjusted for  age, sex, education^2^ | 231 | 1.01 [0.99 to 1.03] | 0.275 | 1.04 [1.01 to 1.06] | 0.001 |
|  | Adjusted for age, sex, education and  pre-stroke GDS^3^ | 231 | 1.01 [0.99 to 1.03] | 0.175 | 1.05 [1.02 to 1.07] | 0.001 |
|  | Fully adjusted^4^ | 226 | 1.02 [1.001 to 1.04] | 0.031 | 1.06 [1.03 to 1.09] | 0.001 |
| WMH  pathology | Unadjusted^1^ | 231 | 2.73 [1.56 to 4.77] | 0.001 | 3.09 [ 1.71 to 5.58] | 0.001 |
|  | Adjusted for  age, sex, education^2^ | 231 | 2.37 [1.33 to 4.23] | 0.003 | 2.54 [ 1.33 to 4.84] | 0.005 |
|  | Adjusted for age, sex, education and  pre-stroke GDS^3^ | 231 | 2.07 [1.14 to 3.76] | 0.017 | 1.94 [0.96 to 3.90] | 0.064 |
|  | Fully adjusted^4^ | 226 | 2.18 [1.14 to 4.17] | 0.031 | 2.86 [1.28 to 6.37] | 0.010 |
| MTA  pathology | Unadjusted^1^ | 231 | 1.95 [1.12 to 3.41] | 0.019 | 1.43 [0.79 to 2.59] | 0.231 |
|  | Adjusted for  age, sex, education^2^ | 231 | 2.13 [1.18 to 3.87] | 0.019 | 1.87 [0.95 to 3.68] | 0.069 |
|  | Adjusted for age, sex, education and  pre-stroke GDS^3^ | 231 | 1.83 [0.99 to 3.39] | 0.056 | 1.35 [0.65 to 2.83] | 0.424 |
|  | Fully adjusted^4^ | 226 | 1.82 [0.94 to 3.52] | 0.074 | 1.36 [0.59 to 3.11] | 0.470 |
| Left hemisphere | Unadjusted^1^ | 226 | 1.80 [1.05 to 3.09] | 0.032 | 1.12 [0.63 to 2.01] | 0.693 |
|  | Adjusted for  age, sex, education^2^ | 226 | 1.74 [1.0 to 3.04] | 0.051 | 1.01 [0.53 to 1.93] | 0.966 |
|  | Adjusted for age, sex, education and  pre-stroke GDS^3^ | 226 | 2.00 [1.12 to 3.59] | 0.020 | 1.11 [0.55 to 2.26] | 0.767 |
|  | Fully adjusted^4^ | 226 | 2.33 [1.26 to 4.34] | 0.007 | 1.72 [0.78 to 3.82] | 0.179 |
| Strategic  infarct | Unadjusted^1^ | 231 | 1.05 [0.44 to 2.51] | 0.91 | 1.13 [0.44 to 2.89] | 0.796 |
|  | Adjusted for  age, sex, education^2^ | 231 | 1.00 [0.4 to 2.48] | 0.999 | 1.17 [0.41 to 3.3] | 0.770 |
|  | Adjusted for age, sex, education and  pre-stroke GDS^3^ | 231 | 0.89 [0.35 to 2.31] | 0.820 | 0.98 [0.31 to 3.08] | 0.975 |
|  | Fully adjusted^4^ | 226 | 1.16 [0.42 to 3.20] | 0.768 | 1.23 [0.34 to 4.46] | 0.749 |

The Odds Ratio (OR) of any NCD or major NCD in the presence of a defined imaging marker is shown in the table above. ORs are reported: ^1^unadjusted; ^2^adjusted for age, sex, and education; ^3^adjusted for age, sex, education, and pre-stroke GDS; ^4^fully adjusted for stroke lesion volume, WMH pathology, MTA pathology, left hemispheric stroke, strategic infarct, age, sex, education and pre-stroke GDS.
