# Supplementary material for: Clinically accessible neuroimaging predictors of post-stroke neurocognitive disorder: a prospective observational study
Source: BMC Neurol. 2021 Feb 25;21:89. doi: 10.1186/s12883-021-02117-8 (PMC7905565; doi:10.1186/s12883-021-02117-8)
Supplement: Supplementary file 4 — Additional file 4: Supplement A4. Definitions of clinical diagnoses, MTA, and Fazekas scale. [file 12883_2021_2117_MOESM4_ESM.docx]

**Supplement A4**

**Definition of Fazekas scale**

The Fazekas scale (Fazekas et al., 1987) is used to assess the extent of white matter hyperintensities on T2-FLAIR MRI sequences. The Fazekas scale is a positive ordinal scale ranging from 0 to 3. With 0 representing no T2-FLAIR hyperintensities and 3 representing large confluent T2-FLAIR hyperintensities in the deep white matter.

The complete Fazekas scale:

- 0 = Absent white matter hyperintensities
- 1 = Punctuate white matter hyperintensities, no confluence
- 2 = Beginning confluence of white matter hyperintensities
- 3 = Confluent white matter hyperintensities

**Definition of MTA score**

The medial temporal lobe atrophy (MTA) scale described by Scheltens (Scheltens et al., 1992) is used to visually assess volume loss of the hippocampus and medial temporal lobe. It was primarily developed for the evaluation of possible Alzheimer disease. The MTA sale is a positive ordinal scale ranging from 0 to 4. With 0 representing no visible CSF around the hippocampus and 4 marked widening of the choroid fissure and temporal horn.

The complete MTA scale:

- 0 = No CSF is visible around the hippocampus
- 1 = Slightly widening of the choroid fissure
- 2 = Moderate widening of the choroid fissure, mild enlargement of the temporal horn
- 3 = Marked widening of the choroid fissure, moderate enlargement of the temporal horn, moderate volume loss of hippocampus
- 4 = Marked widening of the choroid fissure, marked enlargement of the temporal horn, atrophy of hippocampus

## **Definition of clinical diagnosis and risk factor definition**

The following criteria were used in defining clinical diagnoses and risk factors in the Nor-COAST study: Atrial fibrillation (AF) was defined as patients with a pathological ECG recording documenting this heart rhythm, past or present. Hypertension (HT) was noted in patients using anti-hypertensive drugs. Hypercholesterolemia was defined by total cholesterol ≥ 6.2 mmol/L or LDL ≥ 4.1 mmol/L. Diabetes mellitus (DM) was registered when evident in medical records, when the patient used antidiabetic medication, or by a HbA1c ≥ 6.5%. Previous stroke was registered when medical records showed a history of stroke. Stroke severity was assessed by the National Institute of Health Stroke Scale (NIHSS) (Lyden et al., 2001)

Fazekas, F., Chawluk, J. B., Alavi, A., Hurtig, H. I., & Zimmerman, R. A. (1987). MR signal abnormalities at 1.5 T in Alzheimer’s dementia and normal aging. *AJR. American Journal of Roentgenology*, *149*(2), 351–356. https://doi.org/10.2214/ajr.149.2.351

Lyden, P. D., Lu, M., Levine, S. R., Brott, T. G., Broderick, J., & NINDS rtPA Stroke Study Group. (2001). A modified National Institutes of Health Stroke Scale for use in stroke clinical trials: Preliminary reliability and validity. *Stroke*, *32*(6), 1310–1317.

Scheltens, P., Leys, D., Barkhof, F., Huglo, D., Weinstein, H. C., Vermersch, P., Kuiper, M., Steinling, M., Wolters, E. C., & Valk, J. (1992). Atrophy of medial temporal lobes on MRI in probable Alzheimer’s disease and normal ageing: Diagnostic value and neuropsychological correlates. *Journal of Neurology, Neurosurgery, and Psychiatry*, *55*(10), 967–972.
